# Overcoming Whiteness? Romanian Humanitarianism in Sub-Saharan Africa During the 1960s

**DOI:** 10.1177/00220094251393602

**Published:** 2025-11-24

**Authors:** Bogdan C. Iacob

**Affiliations:** Institute for Habsburg and Balkan Studies in Vienna ( 31390 Austrian Academy of Sciences), Austria; Institute of History “Nicolae Iorga” in Bucharest (Romanian Academy), Romania

**Keywords:** state socialism, whiteness, decolonization, race, medicine

## Abstract

In Romania, as throughout state socialist Europe, medical assistance showcased anti-colonial solidarity with newly independent African peoples. The humanism of experts, illuminated through bilateral relations or in campaigns coordinated by international organizations, supposedly revealed their status as better Whites: anti-racist Europeans, considerate of the needs of local populations, and altruistically supportive of their freedom and progress. Yet, socialist humanitarianism struggled to overcome whiteness, understood here as an unmarked category that structured the world and was based on hierarchies of civilization premised upon European superiority. Using the case of Romanian medical missions to Sub-Saharan Africa during the 1960s, I show how socialist commitments to emancipation intertwined with the racialization of the ‘tropics’. I situate Romanian agency in relation to pushback from African actors, while comparing it to other socialist humanitarianisms. This case study exemplifies the White gaze of Eastern European medicine: developmental and clinical evaluations about African peoples both promised modernization and found them regressive and inexorably diseased.

In 1961, amidst Africa's decolonization, a Romanian magazine, *The Woman's Almanach*, featured an article headlined by two photos. One showed a naked, Black child suffering from rickets tended to by a tall, thin White physician dressed in an impeccably white coat (Figure 1). The other zoomed in on the smiling faces of Black children with the caption: ‘The great doctor is coming! His little friends rejoice!’ (Figure 2) The physician in question was Albert Schweitzer, the French-German polymath and Nobel Peace Prize laureate (1952), who was lionized in the socialist camp and beyond for his criticism of nuclear weapons, anti-war activism and humanitarianism towards the victims of colonialism in Africa. The author of the article remarked: ‘Albert Schweitzer demonstrated to Africans that the white man should not be solely associated with colonial lackeys in the West’.^1^ Instead, as another Romanian text claimed in 1965, Schweitzer was ‘the doctor of the blacks’, the symbol for ‘the humanitarian mission of a universal physician in Africa under conditions of extreme primitivism’.^2^ That year, the state socialist regime in Bucharest dispatched five physicians to Conakry, Guinea; another five followed two years later. These experts joined fellow nationals dispatched by Bucharest to various countries in Sub-Saharan Africa via the World Health Organization. Such technical assistance, though modest in comparison with other Eastern Europeans, anticipated a much larger program of exporting Romanian healthcare to the continent during the 1970s and the mid-1980s.

**Figure 1.**
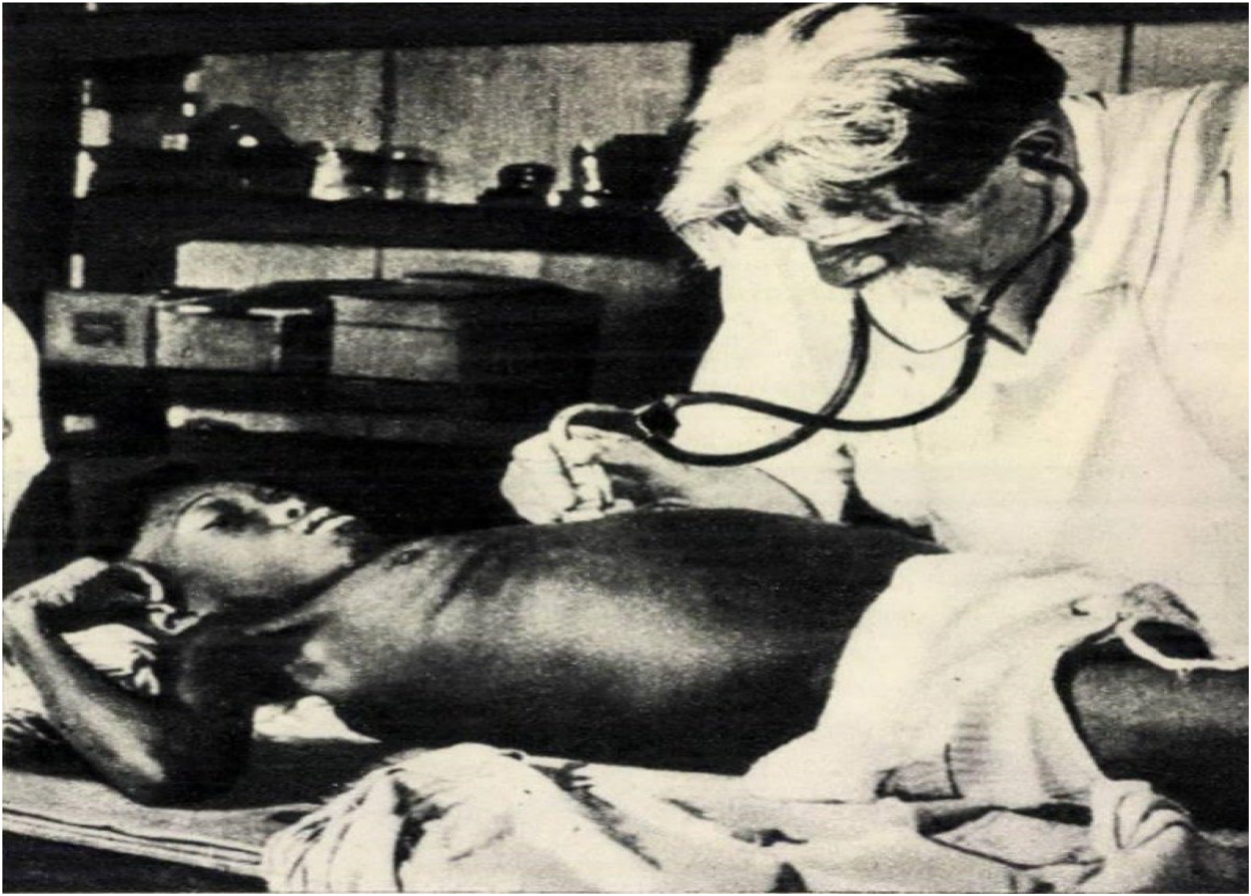
Albert Schweitzer examining a patient. The original caption in Romanian pathologized blackness: ‘Thousands, if not tens of thousands of times, the same scene: the White doctor saves the life of a black’. Source: *Almanahul femeii*, 1961.

**Figure 2.**
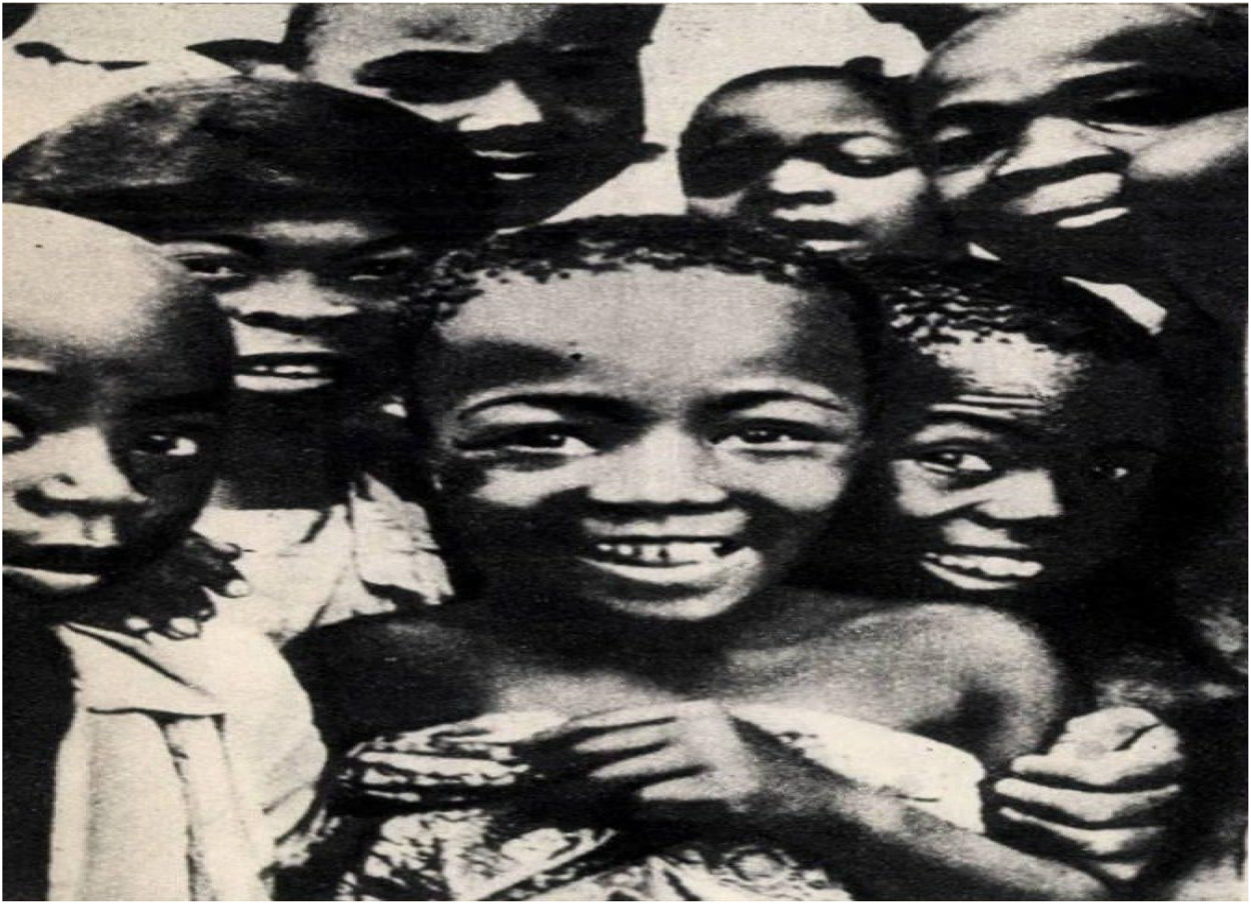
Children at Lambaréné. Source: *Almanahul femeii*, 1961.

Romanian specialists in Sub-Saharan Africa, often called in newspapers and party documents as ‘Black Africa’, embodied the values of socialism at home: they were ‘highly trained and profoundly humane’, lacked ‘mercantile inclinations’ and ‘unflinchingly respected local authorities’.^3^ However, the non-Europeanness of the spaces in which they worked tested their ‘daily humanitarianism’. As one of the doctors remarked, ‘the tropical climate’ fostered ‘debilitating pathologies’ such as ‘infestation with malaria, parasitosis, skin afflictions and endocrinological imbalances’.^4^ Similar to Albert Schweitzer in Lambaréné (Gabon), Romanians struggled to adapt their humanism to what they perceived as ubiquitous contagion in ‘Black Africa’. As anticolonial Europeans (i.e., Whites), the physicians dispatched from Bucharest vacillated between their commitment to the locals’ liberation from disease and socialist perspectives about the modernization of newly independent states. Just like Schweitzer's, Romanians’ humanitarianism intertwined with the racialization of the societies that they cared for.^5^

This article explores how socialist humanitarianism struggled to overcome whiteness, understood here as an unmarked category that structured representations of the world and which was based upon hierarchies of civilization premised on Eurocentric superiority. Using the case of Romanian medical entanglements in Sub-Saharan Africa during the 1960s (Guinea, Ghana, Senegal, etc.), I show how anti-colonial solidarity intertwined with racializing conceptualizations about development, pathology and Europeanness. In Romania and across Eastern Europe, medical assistance showcased the official policy of aiding people from Africa to overcome the colonial legacies of disease, poverty and discrimination. These encounters also produced socialist whiteness, a synthesis of developmental hierarchies and pathologizations of African locales premised upon ideas about European superiority. The anachronic invocation of Albert Schweitzer's ‘ethical colonialism’^6^ symbolized the inability of Romanian socialism to liberate its imagination of the world from the race-centred notions entrenched by the very imperialism that it condemned. Romanians were not an exception among Eastern Europeans in terms of using Schweitzer as a symbol for European, progressive humanitarianism. He was a model in Czechoslovakia and East Germany too, which demonstrates how state socialisms adapted anti-war internationalism to globally legitimize their socialist humanism.^7^

During the 1960s, in Romania, just as in other communist regimes, official discourse used the term ‘humanitarianism’ reluctantly, if at all. For state socialist officials, experts, or journalists, the concept obscured the civilizing mission of colonial empires. It was a compensatory device for the depredations of Western colonialism: rampant disease, lack of education, and economic exploitation. Capitalist humanitarianism was seen as philanthropic and merely a short-term fix. Its self-proclaimed neutrality hid its destabilizing effects on recipient states. Communist governments advocated for ‘socialist humanism’, whose superiority derived from the fact that their countries provided models for overcoming underdevelopment and dependency in the era of empires through state-engineered industrialization and rural uplift paired with high levels of welfare and equality.^8^

The scholarship on socialist humanitarianism and medicine during the global Cold War has been a blossoming field. Authors have highlighted the importance of Eastern European assistance in the national liberation struggle of specific peoples in Asia and Africa.^9^ They underlined the centrality of medical teams from Eastern Europe to the consolidation of national healthcare in the developing world.^10^ Socialist medicine was central to the diplomatic objectives of various regimes as they positioned themselves in a world framed by the Cold War.^11^ Humanitarianism and medicine from state socialist Europe have been situated within the global programs or emergency interventions that were coordinated by international organizations.^12^

However, the existing literature has yet to contextualize postwar Eastern Europe within broader histories of medicine and humanitarianism as tools for empire.^13^ To do so, one has to question the civilizational claims that underpinned state socialist expertise. The emerging research on race and socialism is a means for filling this gap in the literature. Several publications have underlined that Eastern Europeans showcased ‘off whiteness’, historically being in a position of ‘white but not quite’ within the racial orders that were nurtured by modern empires.^14^ Civilizational superiority paired with global imaginations from the region reflected its peoples’ existence at the semi-periphery of a Western-dominated world. Such a position triggered fundamental ambiguities that combined historical subalternity with fantasies about imperial power and the appropriation of colonial knowledge.^15^ During the post-1945 period, anti-colonialism intertwined with ‘socialist racialism’, which was premised on the inclusion of ‘human groups deemed racially different and culturally backward’ into a redistributive, but violent blueprint for development.^16^

The article deploys this conceptual framework to explore the White gaze of socialist medicine (and more generally humanitarianism) using the example of Romanian physicians in Sub-Saharan Africa during the 1960s. Charles Mills once remarked that whiteness was an optic for constructing reality on a racial basis. He understood race beyond biology and skin colour, anchoring it in questions about society, culture and the international order.^17^ In my article, whiteness reflects socialist medicine's commitment to the universalism of a Eurocentric modernity that combined Marxism–Leninism as the ideology of development with pre-socialist notions of civilization, which drew upon colonially embedded knowledge. Romanian physicians racialized peoples in what the regime deemed as ‘Black Africa’ through stereotypes of underdevelopment founded on notions of disease, climate, education, customs, gender and class.

The White saviourism of socialist medicine, as demonstrated by this study, indicated that humanitarian assistance to post-colonial states was tied to debates within the socialist camp and to national representations of Europeanness. During the 1960s, Romania articulated an autonomous foreign policy founded on critiquing, in the context of the Cold War, any form of hegemony from either the West (the United States or former colonial powers) or the East (the Soviet Union in particular, but also China).^18^ Officials and experts from Bucharest combined this anti-hegemonic position with invocations of the country's historical subalternity toward European empires to bolster their relations with states in Africa.

To explore the oscillation between anti-hegemonism and whiteness, I bring together national and international sources. Romanian archives (from the ministries of health and foreign affairs, as well as the Central Committee of the Romanian Communist Party) and newspapers are combined with material from the World Health Organization (mission reports and minutes of the World Health Assembly). I did not have access to archives from the countries in Africa (especially Guinea). I mitigated this problem by highlighting the agency and resistance of local actors in relation to the modernizing gaze that Romanian and other foreign experts deployed. The locale in Africa that constitutes my primary focus is Guinea, but other states south of the Sahara are also discussed.

The article has three parts. The first explores the emergence of Romanian anti-hegemonic stance in relations with countries south of the Sahara, underlying the convergence between Bucharest's critiques of intra-socialist inequalities with African leaders’ anti-colonialism. The second focuses on the experience of physicians in Guinea, highlighting that Romanian whiteness emerged through practices and discourses intertwining ‘African specificity’, Europeanness and ‘tropics’. And the third part expands the analysis of the dichotomy of solidarity and racialization into international programs (mother and child welfare and malaria control) involving Romanian experts in Sub-Saharan Africa.

Africa's decolonization was a transgressive watershed moment for re-imagining the world. While the national liberation of peoples in Asia in the early postwar period set the tone for the reinvention of sovereignty and challenged colonial hierarchies, the breathtaking pace of independence by states in Africa since the late 1950s massively expanded the opportunities for anti-colonial solidarity.^19^ These countries faced staggering obstacles to political, social, economic and cultural emancipation. State socialist regimes hurried to offer development assistance to post-colonial governments. Such efforts were perceived in Eastern Europe and Africa as alternatives to Western aid, which was associated with neo-colonialism. The anti-colonial activism that permeated these trans-regional entanglements went hand in hand with public compassion for the peoples of the continent. Socialist newspapers abounded with accounts about the struggles of African societies to overcome the colonial legacies of poverty, disease and ignorance. Such invocations of Julius Nyerere's famous dictum were humanitarian: they underlined the imperative to help and the global expansion of the community of people who had or who continued to suffer under the yoke of capitalism and empire.^20^

State socialist accounts of decolonization in Africa, and earlier in Asia, converged with anti-colonial activism based upon the idea that human dignity could not be achieved under empires, which had failed to ensure the well-being of subject populations because of social, political and racial discrimination. In February 1961, *Muncitorul sanitar*, the newspaper of the Ministry of Health in Romania, proclaimed that ‘colonial peoples have the right to life and health’. The article detailed the segregation, exploitation and lack of medical personnel fostered by empires in Africa, which had resulted in horrifying epidemics. Invoking debates ongoing at the World Health Assembly in New Delhi, the text stressed that independence was the key response to these crises.^21^ Such narratives linked the plight of people in Africa with past struggles in Romania: ‘ten to fifteen years ago we were not far different from the countries with a backward sanitary situation, despite the high level of [our] medicine’, noted a deputy Minister of Health in Bucharest.^22^

As the above remark signals, anti-colonial solidarity presented Africa as bereft of modernity, which imbued socialist humanitarianism with its own civilizational hierarchies. In Romania and Eastern Europe, officials and experts reproduced what Charles Mills called the chronopolitics of Western, White imperialism: they too ‘denigrat[ed] the inferior times, the obsolete soon-to-be-superseded times, of the non-[Europe]’.^23^ An article about the humanitarian crisis in Congo-Kinshasa caused by the civil war triggered by Patrice Lumumba's assassination condemned colonial medicine. It compiled statistics that showed the omnipresence of diseases and shocking mortality rates among children, as well as racial segregation and the absence of local physicians. Though deemed unreliable, the data were drawn from the reports of the ‘Marcel Wanson’ Institute of Hygiene, a flagship institution of Belgian colonial governance. The figures highlighted the population's ignorance about their plight: ‘thirty percent of women do not know that they should give birth in a birthing home; seventy percent of the respondents do not know that the mosquito is the vector of malaria. The majority of the population does not know about the role of the tsetse fly in the spread of “sleeping sickness”’.^24^ Though anti-colonial and anti-racist, the article did not question the racializing logic of modernization that was implicit in questionable statistics gathered by an institution central to colonial developmentalism in Congo-Kinshasa. By reproducing the latter, the Romanian text endorsed the minimization of African knowledge and constructed an image of backwardness by way of European medicine.

The modernizing gaze that was implicit to socialist humanitarianism often made it blind to the colonizing effects of medicine, which was a broader consequence of Marxism–Leninism – an ideology that endorsed development at breakneck speed across stages in history. Accounts of decolonization, and later expert reports from African countries, mixed paternalism with a racializing subtext. In 1961, a Romanian traveller described a Guinean village: ‘half of the inhabitants were sick [of malaria, leprosy, yellow fever, etc.], and lived nearly naked, their black skin covered by patches of tree bark’.^25^ In this discursive framework, modernity was brought by others: not the ‘missionaries of colonial civilization’ but the apostles of socialist humanism – doctors from the socialist camp.^26^

Such conceptualization of the duty towards suffering bodies made possible the use of Albert Schweitzer as the ideal-type physician for millions of ‘sick [persons] with dark skin’.^27^ He was an example of righteous Europeans using modern medicine for ‘the humanitarian duties imposed by conditions in countries far away’.^28^ Such salvationist paternalism did not go unchallenged by representatives of newly independent African countries. During the 1961 World Health Assembly in New Delhi, when socialist and post-colonial delegates underlined the importance of self-determination for the achievement of health as a human right, experts from Gabon and Senegal criticized the condescension that was inherent to Eastern Europeans’ gaze. They refused to be ‘regard[ed] as children’ and rejected ‘paternalism’.^29^

Notwithstanding public anti-colonial compassion, Romanian authorities showed restraint toward committing aid to African states, especially south of the Sahara. Official documents show that this wariness drew, just as public discourse, on the uneasy mix of paternalism, empathy, ideology and the sheer scale of the problems facing newly independent peoples. Romania was a latecomer to socialist humanitarianism in Africa. While the regime committed significant medical assistance to North Korea and Vietnam during the 1950s, its leadership was slow to emulate ‘brotherly countries’ (Bulgaria, Czechoslovakia, Yugoslavia, or Poland), which dispatched cohorts of experts to hotspots of decolonization, such as Guinea, Ghana, Algeria, or the Congos.

In December 1961, a report of the Ministry of Foreign Affairs noted that Romania was better prepared than other socialist states to offer aid to Francophone African countries because of the widespread knowledge of French among its health workers. It proposed that two or three medical teams be created and placed on stand-by for emergency missions. Yet, the same paper did not recommend substantial aid for three reasons: newly independent states were ideologically unpredictable; post-colonial governments lacked the funds to facilitate bilateral programs; and any assistance from Bucharest had to be a long-term project since no easy fix existed for the challenges faced by African peoples.^30^ A year later, another document suggested an additional explanation for non-involvement: colonial rule had destroyed ‘African civilization without replacing it with anything in return, thus condemning these populations to obscurantism and spiritual poverty’.^31^ The devastation faced by post-colonial societies raised questions about the viability of Romanian interventions. Public representations of Sub-Saharan Africa as a space of disease, poverty and ignorance, which aimed for compassion and signalled the duty to alleviate suffering, echoed in internal state documents, but with the opposite effect: such characterizations stifled significant help for the very people that Romanians empathized with.

Throughout the 1960s, Romanian officials heeded the lessons provided by regimes that had spearheaded state socialist presence in post-colonial Africa. One model was Czechoslovakia, signalled out for integrating aid programs in an ‘active policy’ determined by ‘the need to find larger outlets for its industrial products’, ‘stable and cheap sources of raw materials for [its] industry and domestic consumption’, and by its ability to export goods ‘at world market level’.^32^ At the time, the regime in Prague pioneered state socialist assistance to the continent, trying to develop the expertise necessary for translating aid into long-term cooperation that benefited the national economy.^33^

Another model was Yugoslavia's non-alignment. In this case, Africa was an essential space for the confirmation of the country's new global role and a symbol for Yugoslavs’ claims of being a different type of Whites: modernizers empathetic with post-colonial anxieties about exploitation.^34^ In November 1963, at a meeting in Belgrade, Josip Broz Tito told Gheorghe Gheorghiu Dej, Romania's first communist leader, that newly independent states ‘were still tied to varying degrees and forms of dependence to the metropole […] one had to search for ways to help free them’. Tito advised Dej to approach the matter ‘with great care toward their sensibilities. One must take into account their experience in relation to the whites, thus avoiding anything that would make [these peoples feel] underappreciated’.^35^ Dej took Tito's counsel to heart. A year later, he received the report of a party-state delegation trip to Algeria. In light of a prospective bilateral accord, he warned Romanian officials: ‘we must be mindful of the sensibility of a people that just liberated itself […] We ought to proceed with a lot of tact, modesty, and foresight’.^36^ Still, at the time, the regime in Bucharest did not send significant humanitarian assistance to Algeria. Yet, Bucharest took up two principles from the Yugoslav model: the idea of socialist Whites acting as better Europeans in comparison to their colleagues in the West and the East; and, the theme of ‘selfless readiness’, namely that despite limited human and economic resources, the donor remained committed to assisting post-colonial development.^37^

The Romanian vacillation between the Czechoslovak and Yugoslav models revealed uncertainty about becoming a donor in a post-colonial context and how such a commitment impacted the communist party's national and European status. By July 1966, Romania had opened embassies in only five countries in Africa (United Arab Republic, Algeria, Morocco, Ghana and Guinea). Trade with states on the continent amounted to only 2.2 percent of total foreign trade.^38^ Yet, a breakthrough had taken place in 1964. In April, the communist party issued a declaration concerning its position on the problems within the international communist movement. The document formulated Romania's autonomy in relation to the Soviet Union, proclaiming the pre-eminence of national sovereignty, equality and mutual benefit among socialist regimes, and the imperative of non-interference in domestic affairs. Two months earlier, Dej emphasized the importance of international technological, scientific and cultural openness in order to consolidate Romania's status as a viable global actor.^39^ In October, Alpha Diallo, the Guinean minister of information and tourism (later minister of health), visited Romania and met Dej. The conversation highlighted the new context for Romania's relations with countries in Africa: a common contestation of developmental hierarchies, practices and forms of chauvinism that had been perpetuated by Cold War superpowers and their allies. According to Diallo, Romania and Guinea converged on the common struggle for emancipation: in the present, the two countries stood up to exploitation from great powers, which included both Western states and the Soviet Union; and historically, their peoples suffered ‘the same humiliations’ inflicted by empires.^40^

Romania's liminal position in Europe and the broader socialist camp was the premise for its first significant assistance program in Sub-Saharan Africa. While Sekou Touré distanced himself from the Soviet Union, criticizing Guinea's relationship with socialist countries as profoundly unequal, and characterizing their advisors as ‘depraved’,^41^ the government in Bucharest decided to establish a significant footprint in the country. In a synthesis of the Czechoslovak and Yugoslav models, the communist party premised its aid on anti-hegemonic empathy: the common experience of safeguarding independence in the global Cold War and sharing expertise to overcome the poverty, disease and ignorance that had been fostered by imperial exploitation. Albert Schweitzer was once again the ideal type for such humanist commitment. Just as Romanian doctors arrived in Conakry, one journalist remarked that the ‘great experience at Lambaréné’ symbolized ‘a life-long, active solidarity with the victims of colonialism’.^42^

A year after Alpha Diallo's visit to Bucharest, there were forty Romanian experts (typographers, teachers, doctors, meteorologists and prospectors) in Guinea. Though cost-benefit played a role in contract negotiations, the Romanian regime subsidized such assistance: it continued to pay, at home, the wages of these specialists (in addition to the salaries provided by the Guinean government) and kept their apartments and jobs while they were abroad. Yet, the humanitarian aspect of Romania's first significant assistance program in Sub-Saharan Africa was limited. The Guinean minister of health initially requested twenty-five medical workers. Authorities in Bucharest dispatched a total of ten doctors over five years. Besides their salaries, the Guinean state provided housing, paid leave and covered the cost of plane travel from Romania.^43^

Similar to other newly independent countries, Guinea was a space of multinational medical assistance. Romanian physicians distinguished themselves from other European experts. They affirmed anti-colonial credentials by enacting anti-hegemonic empathy. Doctors claimed that their activities were performed ‘with acute sensitivity toward the fact that administrative matters were deemed internal matters and foreigners’ suggestions incurred significant reservations’.^44^ As mediators between socialist medicine and post-colonial ill-health, Romanians remained apprehensive about local criticism toward displays of overt Eurocentric superiority. Their position was similar to that of Chinese personnel dispatched to Guinea, who insisted on respecting the ‘dignity’ of their hosts and no ‘interference in their internal politics’.^45^

In contrast to their Chinese colleagues, Romanian personnel predominantly worked in Conakry. Authorities in Bucharest were unwilling, unlike their counterparts in Beijing, to commit doctors to rural or remote areas where medical services were scarce. Ironically, even Romanian newspapers highlighted this problem. In a 1967 interview, O. Keyta, at the time the deputy minister of health, criticized ‘Guinean medical graduates’ unwillingness to work in villages, where our greatest problems exist. Malaria, onchocerciasis and parasitosis ravage these [places]’.^46^ Yet, Bucharest predicated its dispatch of physicians upon their employment in Conakry, which reinforced the neocolonial reality of a healthcare system geared towards urban services with limited reach into the countryside.

Around the same time, Osei Owusu Afriyie, the Ghanaian minister of health, inquired about the possibility of Bucharest dispatching a team of eighteen medical workers. Afriyie specified that they would work in the countryside, in the northern territories. The government in Accra sought to extend services in a region historically underserved and peripheral.^47^ Each doctor would practice both curative and preventive medicine. Housing and wages proposed by the host country were worse than those negotiated with officials in Conakry.^48^ A document from the Ministry of Foreign Affairs in Bucharest lambasted the proposal as ‘unacceptable and outright offensive’.^49^ Anti-hegemonic solidarity had its limits: Romanian doctors – embodiments of socialist medical modernism – would not be demoted to the status of rural physicians at the periphery of an African state. The communist leadership's decision might have also been tied to the Ghanaian ambassador in Bucharest's protest against racially motivated attacks against students from Africa. These events embarrassed the regime and took place in the Romanian capital in late 1965 and early 1966. Socialist anti-colonial solidarity dissipated when its self-proclaimed colour-blindness was contested.^50^

The Romanian regime's focus on urban health care in Sub-Saharan Africa was paradoxical. It contrasted with Romania's self-representation within the socialist camp and towards newly independent states. At the meeting of socialist ministers of health in 1961, the delegation from Bucharest boasted that 36 per cent of medical cadres worked in the countryside. In the correspondence with his counterpart from Egypt, the minister of health underlined the extensive rural medical coverage in Romania, which was a symbol of the country's post-1945 modernity.^51^ Nevertheless, in Sub-Saharan Africa, officials in Bucharest did not work with the same urban–rural scale with a view to health as they did in Romania. While noting the potential for modernity in Guinea and other states, socialist humanitarians considered such prospects limited by underdevelopment, disease, poor education, or obscurantism in the countryside. The exoticism and wildness of societies in Africa constituted the backdrop for Romanian whiteness.

Romanian physicians constructed Guinean society - an exemplum for Sub-Saharan Africa – as the ‘Other’ in relation to their Europeanness. They did so by referring to the role of state, class struggle, culture, climate and disease. Such othering fuelled racialization: ‘a paternalistic vision of socialist civilizing mission to aid benighted African blacks stuck in backwardness’.^52^ Specialists saw themselves as anti-colonial Europeans (i.e., Whites) acknowledging the struggles of a recently liberated people. Yet, the social, economic, cultural and environmental otherness of Guinea generated poor health indicators. Romanian, and more generally, socialist doctors fused terminology concerning ‘tropical pathology’, ‘climate unfavourable to Europeans’ and under-development to define ‘Black Africa’ and confirm their implicitly White civilizing mission.^53^

A report of the Romanian Embassy in Conakry issued shortly after the arrival of the first team noted its members’ initial shock with post-colonial conditions: ‘their first contact with Guinean reality, which is absolutely different than Romania, has generated dissatisfaction and disparaging comments’.^54^ Subsequent Embassy and expert evaluations revealed that Romanians were looking in on, and down upon, ‘African specificity’ (*specificul african*) considered alien and alienating. Such ‘specificity’ comprised the ‘tropical climate’, which made the adaption to local conditions difficult; and, a ‘tropical pathology’ characterized by ‘extensive parasitic infection and contagion’. Other features listed by Romanian physicians were: poor hygienic conditions in hospitals, lack of medicine, serums, plasma and blood for transfusions; and, the simultaneity of general and specialized medical care because of the limited infrastructure, which generated extreme workloads for the team.^55^

The reports’ emphasis on ‘tropical’ otherness differed from official discourse in Bucharest. Romanian authorities preferred to talk about medical challenges faced or posed by ‘countries with weather difficult to bear’ [for Romanians/Europeans].^56^ During the 1960s, Romania, in contrast with regimes in Czechoslovakia, Poland or the Soviet Union, mostly shied away from notions such as ‘tropical medicine’ or ‘tropical diseases’, which it associated with colonialism and its legacy. Until the mid-1970s, for financial reasons and because of the country's slower engagement with the Global South, there was no attempt to create a socialist specialism in tropical diseases. Yet, in line with the reports from Guinea, the government underlined the specific pathology and the dangers to Romania and its citizens originating from ‘countries with weather difficult to bear’. Between 1965 and 1966, the ministry of foreign affairs in collaboration with the ministries of health and internal affairs designed a program for specialization in and control of ‘highly infectious diseases’ (cholera, plague and smallpox) at institutions in India and Egypt. The aim was to create the infrastructure and personnel necessary for protecting Romania from epidemic dangers triggered by its current and prospective relations with states in Asia and Africa.^57^

In Guinea, similar concerns about pathology characteristic to ‘countries with weather difficult to bear’ guided the selection of the Romanian team's leader. Traian Roșca, a specialist in internal medicine at the Emergency Hospital in Bucharest, had worked in Afghanistan, treating oilmen employed by a Soviet-Afghan company (Tofhassate-Petrol). The chief physician of the mission, showcasing Romanian anti-hegemonic solidarity, acquired his expertise through pan-socialist cooperation. Between 1962 and 1963, in collaboration with Soviet, Afghani and Czechoslovak doctors, he gathered experience in the management of epidemics (cholera, smallpox, malaria), in emergency surgery under poor conditions, and in advising on hospital construction and sanitary education.^58^ He brought this knowledge to Guinea.

Roșca's transfer from Afghanistan to Guinea combined with state policies toward ‘countries with difficult to bear weather’ reveals the practical perspective assigned to the tropics, despite official reluctance toward such terminology. For Romanian experts, the tropics signified climate determinism paired with notions of underdevelopment, scarcity and pathology. The challenge for Romanian physicians was formidable because, according to an Embassy report, Guinea, due to its marginalization in the French empire, had ‘an impoverished social-cultural and economic infrastructure’, compared to Senegal or Cote d’Ivoire.^59^ The ‘African specificity’ of Guinea, partially determined by colonial legacies, placed the country and its society on the margins of socialist modernity.

The practical discourse about ‘tropical’ Guinea (and by extension ‘Black Africa’) echoed the uneasiness of Romanian, and more generally Eastern European experts, with the country's non-capitalist path to development. The Soviet Union, along with other members of the socialist camp, envisaged for Africa a modernization founded on the consolidation of and investment by the state sector, the creation of agricultural cooperatives, and planning without abolishing private property or market economy. Communist regimes provided assistance and credits to reinforce local governments and limit as much as possible the influence of international private capital.^60^ In early 1966, Traian Roșca remarked the paradoxes of Guinea's transition. Upon visiting a pineapple plantation, he noted the small participation of the state in the enterprise, which remained under the control of former French patrons.^61^

In health care, Guinea's non-capitalist development was the backdrop for Romanians’ insistence on local failures according to socialist medical criteria. Without denying the promise of modernity, they positioned themselves as socialist civilizers assessing the ability of the state to liberate Guineans from disease. They deplored the fees that citizens paid for services. They even presented themselves as exploited benefactors: ‘Fees for hospitalization, tests and treatment are high. Every physician brings to the hospital a daily income equal to his monthly salary. … each of us treats a number of patients that is three times higher than the contractual norm’.^62^ The Romanian Embassy tempered their ideological zeal: an official noted that hospitals charged fees to survive the recession, adding that the claims about the profit incurred were exaggerated.^63^ Despite a commitment to non-interference in domestic affairs, doctors sometimes lambasted authorities for limited investment in medical infrastructure and a lack of interest in sanitary education. Paradoxically, considering Bucharest's insistence about Romanian experts’ presence in Conakry, the physicians complained about poor connections with the region surrounding the capital, which prevented them from tracking patients.^64^

Another element of the dissonance was the class character of local health care personnel. Physicians criticised Guinean colleagues for their ‘the mentality of capitalist practitioners.’ Local staff lacked the competence and morality of socialist physicians; they were mostly educated in France, ‘satisfactorily for the requirements here’. Their class-based behaviour allegedly gave way to racist attitudes toward Romanians.^65^ Similar to other Eastern Europeans, the doctors found it difficult to cope with the fact that ‘their white skin bore the mark of European imperialism, they thought only Western Europeans should really have to bear’.^66^ Guinean doctors also headed the specialized wards of hospitals in Conakry; Romanians had to abide by their authority. Such a situation could foster tense relations since Romanians considered that some of their colleagues were no better than ‘mid-level cadres’ in Bucharest. The uneasiness toward Guinean staff could also be inferred from complaints about the putative ‘absence of scientific activities’: there were no medical society, no conferences or specialized periodicals. Romanian claims about a putative ‘local resistance toward research’ were exaggerated. Conakry hosted multiple WHO expert missions tasked with studying disease control, basic health care, training or pharmaceutics.^67^

Such critique combining class, academic and racial motifs highlighted that Guinea's non-capitalist modernization remained dissonant from European-style socialism. Romanians were hardly alone in questioning post-colonial progress. Austin Jersild has shown that Eastern Europeans ‘attributed the failures of socialist collaboration to indigenous culture and practices and a local propensity for […] African corruption […] a product of poverty and the colonial experience’.^68^ Moreover, by lambasting local expertise, Romanians and Eastern Europeans emulated colonial suspicions toward native medical workers, often suspected of mere mimesis rather than acculturation to Western civilization.^69^

The social-economic framing of ‘African specificity’ as non-capitalist (under)development – the negative counterpart to socialist modernity - mirrored the pathologization of peoples and spaces in ‘Black Africa’. Romanian doctors in Guinea echoed representations in Bucharest about the epidemic peril latent in ‘countries with weather difficult to bear’. According to a report of the first team, ‘hospitals do not have separate sections for contagious diseases; paediatrics and internal medicine wards hospitalize cases of typhus, yellow fever, dysentery, malaria, poliomyelitis, parasitoses, hepatitis, eruptive diseases and sometimes even tuberculosis or leprosy’.^70^ Widespread infectious diseases, non-systematic care and unsanitary habits indicated poor hygienic education and insufficient attention to prevention, two central aspects of socialist medicine. These representations echoed colonial narratives about pathological environments and local peoples as immature native germ carriers. Their paternalism harked back to the lionization of Albert Schweitzer's civilizing exploits in Lambaréné. One article indicated Schweitzer's adaptation to native customs: ‘the black, who has a clan mentality, will not agree to hospitalisation without one or two members of his family’.^71^ Similarly, the first team in Guinea noted that ‘families visit at any hour … meals are served together, patients and their families, directly on the floor’. Disease, insalubrity and retrograde sociability added to ‘African specificity’ (i.e., blackness).

Such clinical and sanitary pathology could be eradicated by socialist, rational Romanians (Figure 3). They brought ‘better organization of medical practice, a more rational use of local resources and sanitary education’.^72^ Traian Roșca created an ideal type for the Romanian humanitarian dispatched south of the Sahara: ‘to work here, doctors must have prior training in tropical pathology’, ‘be in perfect health’ and ‘with iron-cast morale’ to overcome the difficult climate and socio-economic conditions.^73^ Such commitment to bringing modernity to a ‘tropical’ space was similar to earlier prescriptions for colonial doctors. During the 1900s, Belgian medical administrators insisted that physicians stationed in Congo had to be mature but not too old, with extensive experience, in good health, and with a ‘robust constitution’. They required ‘excellent morals, and an energetic and virile character, that does not get daunted or discouraged, not by sickness and not by boredom’.^74^ Socialist and colonial doctors converged on the belief that they brought the universal benefits of European medicine to underdeveloped, diseased, Black people.

**Figure 3.**
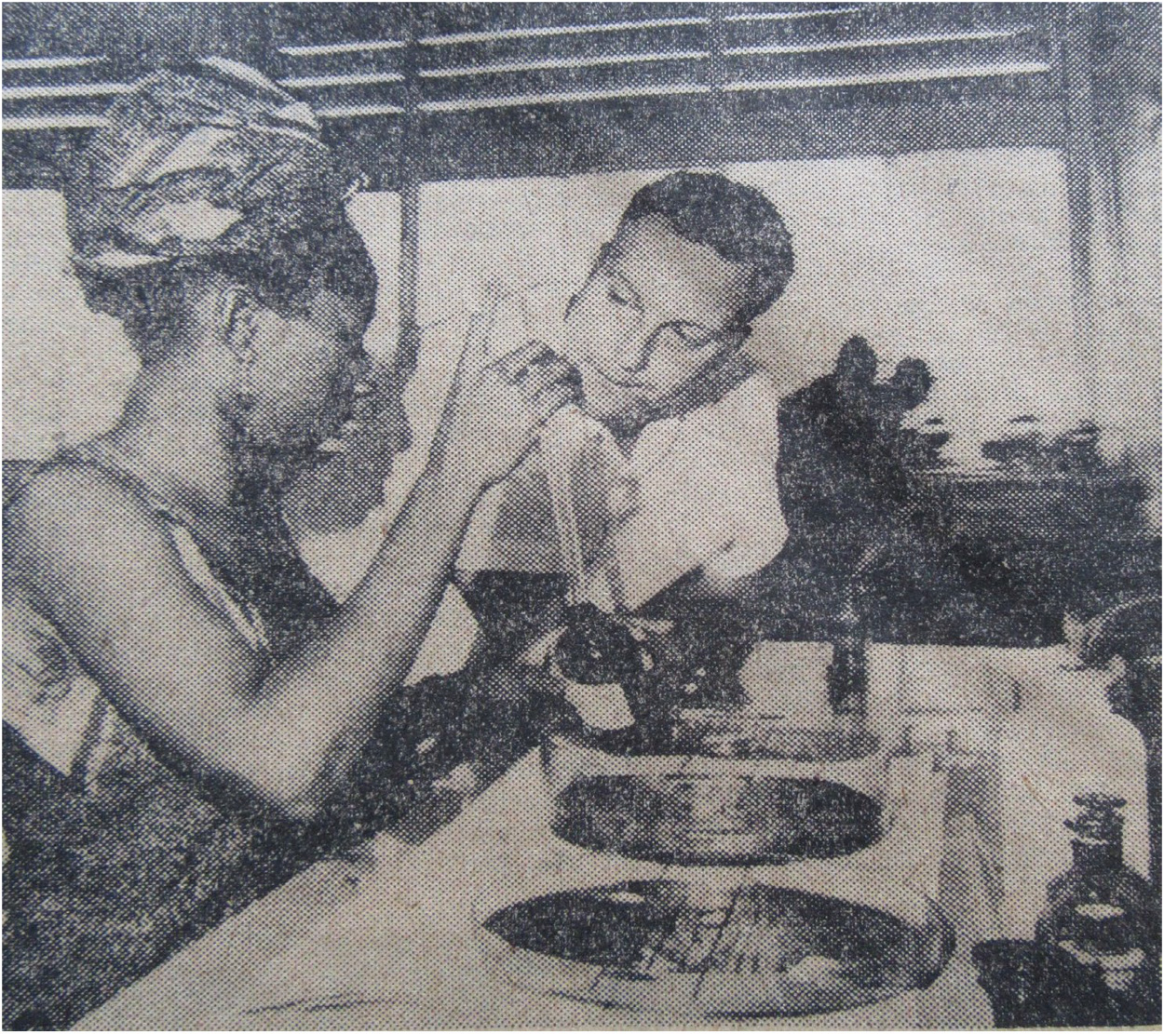
Socialist doctor and African student. The image showcases the emancipatory effects of Romanian-Guinean health bilateralism. It also underlines the civilizational hierarchies implicit to this relationship: the Eastern European doctor in white coat teaches a Guinean woman in traditional garb. Source: *Muncitorul sanitar* 1969.

Guinean officials countered the civilizational claims of socialist experts. Alpha Diallo condemned ‘foreign physicians’ for their lack of enthusiasm and professional ethics, accusing them of betraying their commitment to aid Guinea. He branded their critiques of the less-than-ideal working conditions in the country as neocolonial.^75^ Local authorities rejected any assessments that reproduced narratives about a savage continent incapable of self-governance. They insisted that foreign experts ought to be sensitive to the local conditions for socialism, which proved to be the reason for Sekou Touré's break first with the Soviet Union and later the United States. In January 1962, he warned Anastas Mikoyan, the first deputy chairman of the USSR Council of Ministers, that ‘we are against paternalism in any form and give priority to the dignity of our people over any aid’.^76^ Counselled by the Embassy staff in Conakry, Romanian specialists carefully balanced their civilizing claims with proving their humanitarianism in contrast to other Europeans.

Elements of Romanian representations about the ‘African specificity’ could be repurposed to symbolize the promise of non-capitalist modernization. In February 1966, Alpha Diallo and Traian Roșca visited the leper colony in Forécariah, which was located 90 km from Conakry. For Diallo, the location epitomized post-colonial emancipation, showcasing the integration of lepers into the liberated society. Tackling leprosy by way of preventive and curative measures symbolized the revolution implemented by Sekou Touré's Democratic Party.^77^ WHO materials highlighted the same progress witnessed by Roșca. In 1965, the organization dispatched Étienne Montestruc to assess the Guinean anti-leprosy program. The director of the Pasteur Institute in Martinique and former coordinator of disease control programs in the French colonies, Montestruc recommended the systematic isolation of patients. His suggestion was rebuffed by officials from Geneva, who underlined the efficacy of the Guinean integrative approach.^78^ In Forécariah, Roșca did not only observe the Guinean revolution in action. He also emulated Albert Schweitzer, whose fame was linked to caring for lepers. Roșca's engagement with this disease was a rite of passage confirming his humanism.

Additionally, Constantin Ionescu, a surgeon, highlighted the technological potential of post-colonial Guinea. He published an article about the Institute of Applied Biology in Kindia. The institution provided venom and serums to West Africa and Europe. For Ionescu, it was a symbol for ‘the contemporary efforts in newly independent African states to showcase as much real knowledge about the nature of these regions so that they would be able to master the forces of nature’.^79^ The Institute used to be a node in the colonial network of Pasteur Institutes. The article highlighted the Africanization of formerly imperial infrastructure. It echoed WHO reports about the Institute in Kindia, a regional centre for the production of lyophilized vaccine essential to vaccination against smallpox across West Africa.^80^

Yet, ‘tropical’ Guinea, as a symbol for ‘Black Africa’, remained a perilous space: not only because of its pathology and underdevelopment, but also because it endangered the intellectual and moral constitution of socialist physicians. They requested scientific and party literature to remain up-to-date with the academic and ideological achievements engineered by the regime in Bucharest. Embassy officials too warned about neurosis-like symptoms among physicians caused by the difficult climatic and professional conditions.^81^ These accounts gave a socialist twist to their archetype of European humanism – Albert Schweitzer. In Lambaréné, Schweitzer agonized about his spiritual integrity as a European (i.e., White) living among people whom he deemed to be without culture, like ‘children’ requiring ‘higher authority’.^82^ Echoing his paternalism, Romanians affirmed superiority to counter their degeneration as socialist, European (i.e., White) civilizers.

Sub-Saharan African countries were not just spaces of intersecting national medical missions. Newly independent states were sites for programs developed in collaboration with international organizations. Romania showcased its anti-colonial humanitarianism by taking on a significant role in such programs. These activities also showed how socialist assistance to post-colonial peoples became entangled with racializing practices and narratives. I focus here on pronatalism and malaria control as means for nation-building. First, Romanian experts used global debates about reforming health in Africa to advocate for the expansion of the state sector. Second, they engaged in quasi-eugenic approaches to maternal and child care, and in the colonially-inspired discourses about acquired immunity to malaria south of the Sahara. The overarching theme was the vitality of the anti-colonial nations. The topic brought together socialist and post-colonial societies, but it equally affirmed a civilizational gap between Europe and Africa.

The first instance of such internationalization predated any bilateral assistance. In November 1960, a month before the Declaration on the Granting of Independence to Colonial Countries and Peoples was adopted at the UN, the Romanian Ministry of Health organized a workshop in Bucharest entitled ‘Protecting Mother and Child’ under the umbrella of the Women's International Democratic Federation (WIDF). The latter was a pro-Soviet organization that agitated for peace, anti-imperialism and women's rights. The event gathered together Afro-Asian participants (Mali, Guinea, Cameroon, Congo, Togo, Tunisia, Lebanon, Iraq and Indonesia). It took place during a period of internal criticism within the WIDF concerning its predominantly White leadership.^83^ Paradoxically, the welcoming presidium exclusively included White women: Romanian officials along with Odile Arrighi, the WIDF secretary, a French communist.

The proceedings proclaimed that improving the health of mothers and their children was a key indicator of progress. Newspapers and local participants described Romania as a guide for Asian and African women to ‘eradicate the heavy legacy of the past: misery, lack of culture, and disease’.^84^ Indeed, the Malian delegate, Rokiatou Sow, declared that the seminar, along with the hospitals that she visited, represented ‘a genuine school where one can see with clarity the great achievements that a free people can make in a brief historical period’.^85^ There were discussions about the dissolutive impact of abandonment, prostitution or lack of education on newly independent societies. Such arguments reflected the intersection of post-colonial and socialist modernist anxieties. Afro-Asian speakers characterized these phenomena as colonial legacies and developmental hurdles for state-building. In the Romanian case, displacement paired with fluid family and gender relations were associated with the breakneck pace of building socialism as well as with the liberalization of abortion, which was legislated in 1957. In 1962, one of the factories (workers here were mostly women) visited by the Afro-Asian participants during the workshop was the site of the first major fertility survey in socialist Romania. Its findings elucidated the social and economic factors that influenced the reproductive behaviour of employed women and warned about low-birth rates.^86^ The issues discussed at the WIDF workshop anticipated the instrumentalization of pronatalism in Sub-Saharan Africa and Romania, which gave a eugenic tenor to humanitarian concerns about maternal and child health. Indeed, one of the articles about the seminar opened with a quote from Gheorghe Marinescu, the founder of the interwar Romanian Society for Eugenics and the Study of Heredity, deploring ‘the non-rational approach’ of puericulture in pre-socialist times.^87^

The star of the Bucharest seminar was Loffo Camara, a senior member of the Democratic Party in Guinea and a trained midwife (Figure 4). Romanian materials identify her as ‘the most important personality’ at the proceedings, not least because she symbolized Guinea's trail-blazing status in Africa's decolonization.^88^ A year later, Camara became the minister of social affairs. She negotiated with authorities in Bucharest the dispatch of physicians to Conakry, but the discussions extended until late 1964 and were taken up by Diallo. Camara's position became tenuous because of her criticism of Sekou Touré's authoritarianism, and in 1970, she was arrested and executed.

**Figure 4.**
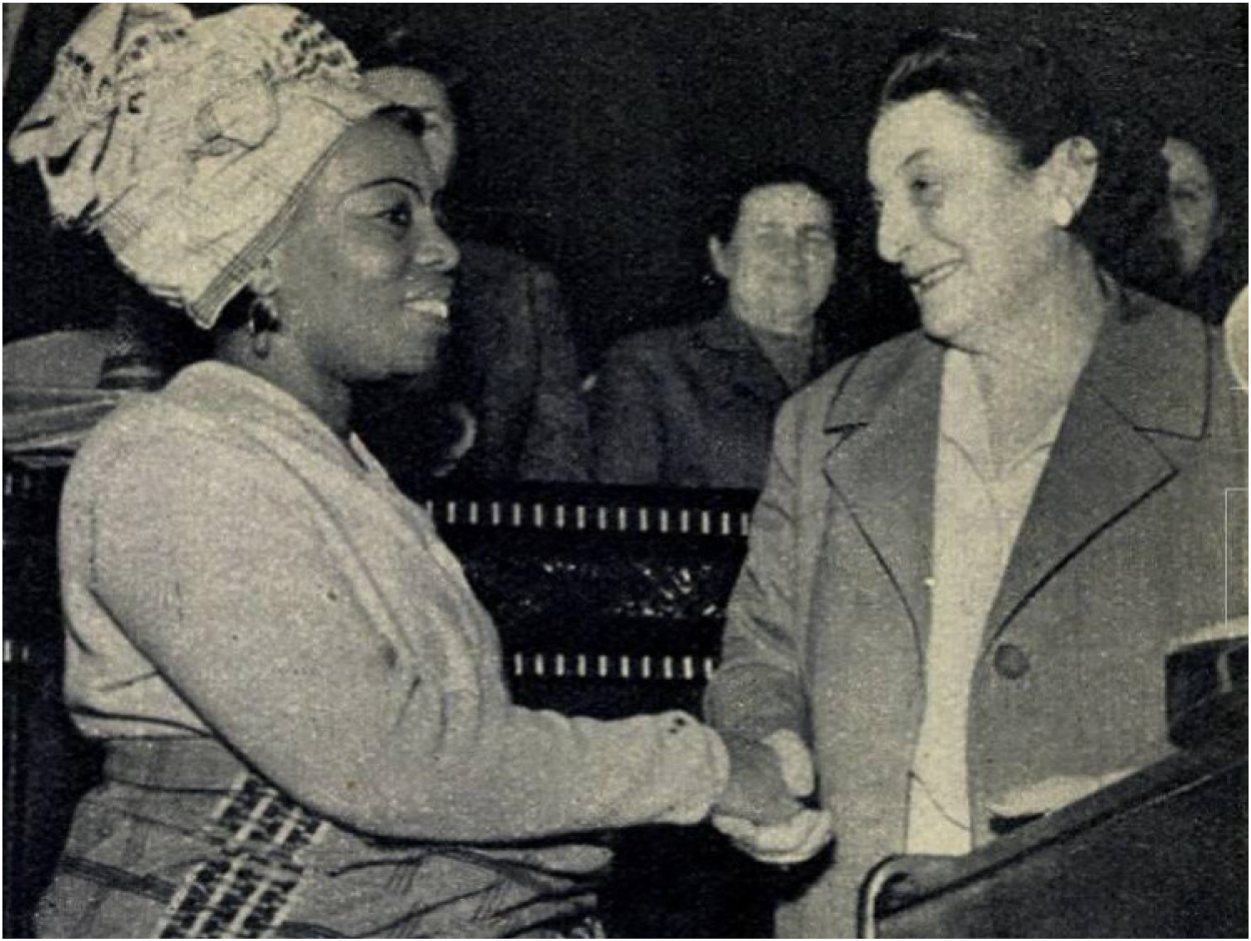
Loffo Camara and Maria Rosetti, the president of the Romanian Women's National Council at the ‘Mother and Child’ seminar. Source: *Femeia* 1960.

Nevertheless, Loffo Camara secured an increase in Romanian scholarships for Guinean medical students, which allowed officials in Conakry to learn about socialist technological breakthroughs. In March 1966, Diallo travelled to Romania and inquired about purchasing a newly developed vacuum suction apparatus recently tested by two gynaecologists in a hospital in Bacău (300 km from Bucharest). Similar to Romanian experts, Diallo saw this technology as crucial for safer births. Notably, authorities in Bucharest advertised the apparatus at a time when the party-state was drafting the decree banning abortion.^89^ Guineans and Romanians converged on their common concern regarding the biological viability of their nations.

However, anxieties among Romanian humanitarians about the future of newly independent nations could also reinforce perceptions about the inexorable pathology of ‘tropical Africa’. In 1955, the WHO launched a global program for malaria eradication. The initiative focused on Latin America, the Western Pacific and Southeast Asia. As decolonization swept through Africa, some states on the continent were included through pre-eradication programmes. The latter pursued malaria control to build capacity but were disconnected from national healthcare. Romania stood out in the WHO campaign: the organization used the eradication of the disease in this country (1963) as a model for reforming its practices, shifting from the near exclusive focus on insecticide spraying and quinnization to multisectoral control programs integrated into basic health services. In May–June 1965, the WHO dispatched Gheorghe Lupașcu, the deputy director of the Institute of Microbiology and Epidemiology in Bucharest, to Senegal, Sierra-Leone, Togo, Nigeria, Tanzania and Madagascar to assess these states’ policies for the treatment and prophylaxis of malaria. Notably, Lupașcu employed the colonial terminology ‘republics in tropical and equatorial Africa’.

Still, echoing socialist approaches, he insisted on the role of governments in coordinating curative and preventive measures. He insisted on the expansion of basic health services in the countryside as essential to anti-malaria programs. Yet, Lupașcu remarked that ‘African specificities’ threatened any progress against the disease. First, he drew attention to these states’ rural character and the epidemic potential of nomadism: pastoralist populations (such as the Fula people in West Africa) carried malarial strains across borders. His remarks echoed eradication in Romania: eradication had been mixed with collectivization and the forced settlement of nomadic communities historically deemed non-White (e.g. Roma or Muslim pastoralists).^90^ Lupașcu's veiled invocation of social engineering allowed for the pathologization of blackness: ‘the European is taken aback by the diversity of human types: tall, well-built people, with skin ranging from black, black-ash to brown […] Unfortunately, the eye often catches the depressing sight of children and adults mutilated by disease, as their community cannot provide proper care’^91^

Second, Lupașcu highlighted the infrastructural and budgetary weakness of Sub-Saharan states, which prevented ‘the application of scientific measures [for disease control], even on a very reduced scale, while awaiting the development of basic health services’. Even champions in the government-subsidized treatment and prophylaxis, such as Senegal or Madagascar, faced escalating costs that threatened the long-term viability of their programs. In all six cases, Lupașcu underlined that the preventive and curative coverage of the population was ‘proportional to the energy shown by the responsible organization as well as the development of the distribution network (medical or voluntary) and of general discipline’ [Figure 5].^92^

**Figure 5.**
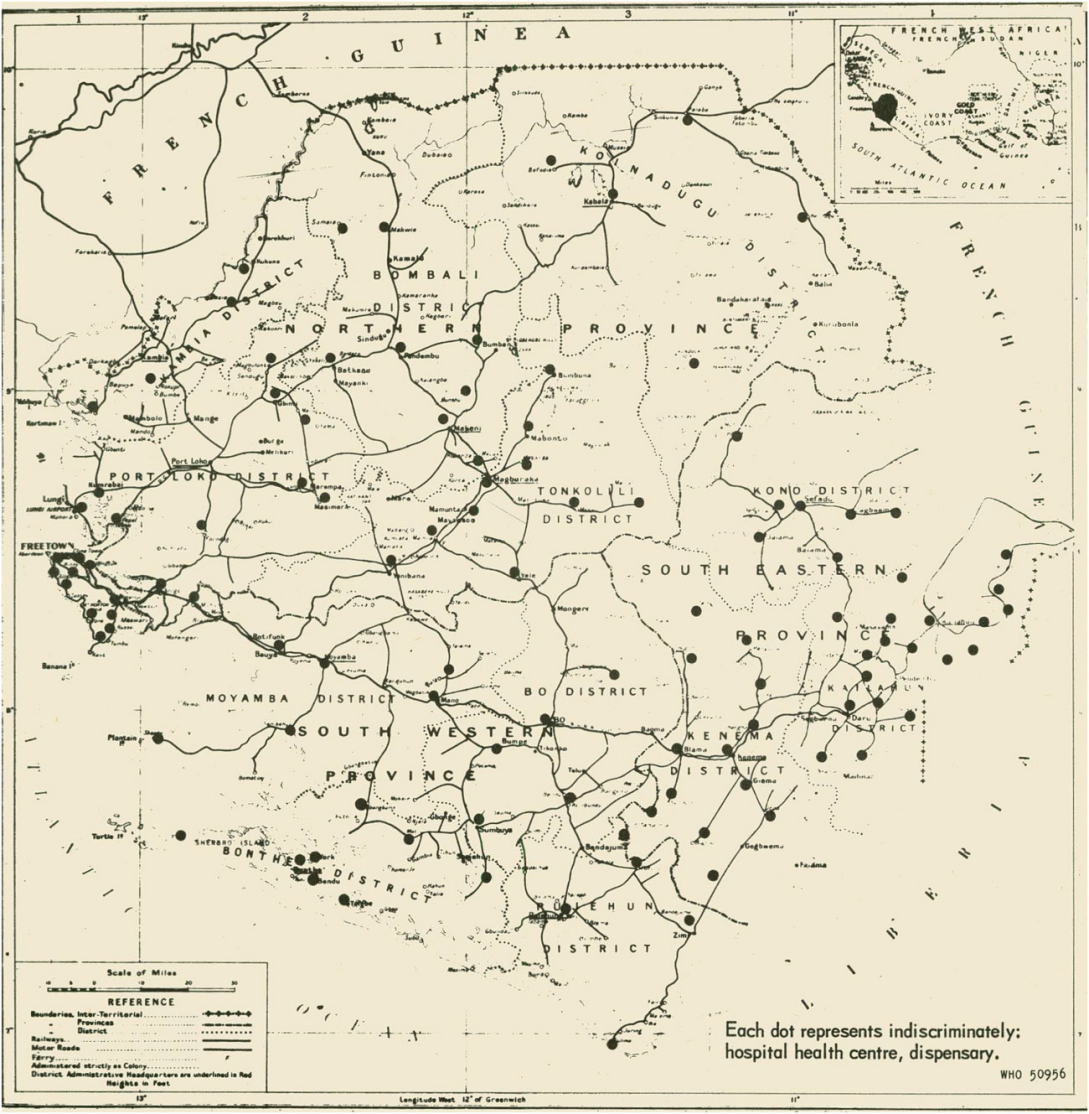
This map of Sierra Leone was included in Gheorghe Lupașcu's report. It combines colonial and post-independence geographic descriptions of West Africa. Source: WHO007/Malaria1/EXPCO/12th/2.

His pessimistic outlook on post-colonial governance set the ground for an ominous warning: anti-malaria programs in ‘tropical Africa’ potentially disrupted local populations’ acquired immunity to certain strains of the disease. Tellingly, newspaper coverage in Romania of Lupașcu's WHO mission did not include this discussion of immunity to malaria south of the Sahara. This fact indicated the communist regime's uneasiness during the 1960s to publicly address a topic that had been racially instrumentalized by colonial medicine. Yet, in his WHO report, Lupașcu echoed postwar reservations, mostly among British and Dutch colonial experts, against the enthusiasm of North American malariologists and post-colonial officials who considered, first, the discovery of DDT (and other insecticides), and, second, the development of anti-malarial drugs (e.g. chloroquine) as magic bullets for the interruption of malaria transmission in hyperendemic regions (deemed ‘tropical’).^93^ Lupașcu advised against ‘any use of residual insecticides for malaria control’, a practice popular in postwar French and Portuguese-ruled Africa. He opposed this approach because it generated resistance among the disease-carrying mosquitoes. Instead, he emphasized that ‘every possible effort should be made to ensure the treatment of infants and young children, aged from 0 to 2, or even from 0 to 5, who are the most exposed because they are without immunity’. In the case of adults, he only recommended the treatment of fever cases for the reduction of the level of infection, so a balancing of curative intervention with the preservation of acquired immunity.^94^

Lupașcu's opinions about immunity, post-colonial governance and health care – a mix of socialist medicine and arguments formulated in colonial contexts - effectively proclaimed an unbridgeable epidemiological gap between Africa and Europe. He concluded in an interview in Romania: ‘on the African continent … we are far from catching a glimpse of eradication, as it has been done in Europe’.^95^ Yet, the plight of people riddled with malaria validated the European prestige of socialist expertise. After returning from his WHO mission, the British Royal Society for Tropical Medicine and Hygiene nominated Lupașcu among its members. Upon receiving the distinction, he argued that the cooperation between the Institute of Microbiology in Bucharest and the London School for Tropical Medicine, which was facilitated by the WHO, benefited peoples from ‘tropical Africa’.^96^ In the second half of the 1960s, as Romanian anti-hegemonic foreign policy gained steam, the country's experts joined the modernizing, pan-European (i.e. White) mission to heal ‘tropical’ (i.e. coloured) peoples, whose states proved unable to care for them. Yet again, the ghost of Albert Schweitzer haunted socialist humanitarianism.

Romania's medical assistance in Sub-Saharan Africa during the 1960s demonstrated to the communist leadership that healthcare was a useful soft diplomacy tool during the Cold War. After April 1972, when Nicolae Ceaușescu returned from his first tour of the continent (he visited eight states), Romania gradually dispatched thousands of medical workers to countries in Africa. Four years later, the number of experts hired by the WHO reached a high of twenty-seven specialists (third among Eastern Europeans); they mostly worked in Africa. The regime's increase of medical assistance reflected Romania's accession to the group of 77, a coalition of developing countries lobbying for a new international economic order. However, bilateral programs usually had a different profile from the trial-run in Guinea: they sought hard-currency or facilitated profitable infrastructural contracts for Romania.

Romania's aid to countries in ‘Black Africa’ reveals the limits of socialist medicine as alternative to Western humanitarianism. Anti-hegemonic, modernist optimism about Africa co-existed with Romanian experts’ propensity for paternalism, which was rooted in Marxist-Leninist developmentalism and drew on particular Western claims of civilizing mission at the ‘tropics’, personified by Albert Schweitzer. However, by zooming in beyond these claims of superiority, one also finds post-colonial agency. Socialist humanitarianism was co-produced: physicians adapted to local actors’ demands and conditions in Africa.

Romanian doctors went to great lengths to affirm the alternative nature of their commitment to the health of newly liberated peoples. They extolled the state's educational and decision-making role in medical care, advocated the expansion of basic services into the countryside, critiqued economic inequalities and insisted on the deleterious impact of colonial legacies. Echoing the Romanian regime's self-representation, they emphasized sovereignty and self-determination in health governance.

Simultaneously though, Romanians epitomized socialist uneasiness with non-capitalist development in Africa. Upon discussing the problems of post-colonial medicine, they talked about ‘African specificity’. They diagnosed the latter through conceptualizations about ‘tropical’ backwardness, pathology and climate. By insisting on their status of Europeans bringing modernity to ‘Black Africa’, Romanian humanitarians fell back on racializing themes. They deployed a socialist White gaze that relied on epistemic narratives constructing African (i.e. Black) peoples against a Eurocentric civilizational gird that carried the promise of modernization and emancipation, but it often found local societies lagging, regressive and inevitably diseased.^97^ The hybridity of this gaze – partly socialist, partly expressing longer histories of medicine as tool for empire – points to a broader reality of the Cold War period: the resilience of race at the core of humanitarian politics beyond ideological divides.

## Notes

1 I wish to expressed my gratitude to Catherine Baker, Anikó Imre and James Mark. They had a formative impact on my understanding of the relationship between global histories of race and empire, and the trajectory of Central and Southeastern Europe during the twentieth century. Our collaboration for the volume *Off White: Central and Eastern Europe and the Global History of Race* (Manchester 2024) was essential for my work on the current article. I am also grateful to the two anonymous peer-reviewers. Their suggestions and criticism helped me sharpen and polish the article's main arguments. D. B., ‘Savantul din junglă’, *Almanahul femeii*, 1961, 74–5.

2 V. Bologa, ‘Albert Schweitzer’, *Tribuna*, 16 (1965), 12.

3 ‘Informare: grupul de specialiști, cadre didactice și medici detașați în Republica Guineea’, 10 February 1966, MSPS [Ministerul Sănătății și al Protecției Sociale] Secretariat Relații 1966 vol. II, 197–6. As early as 1960, the term ‘Black Africa’ was central to the regime's foreign policy plans in Sub-Saharan Africa: ‘Notă cu privire la deschiderea de oficii diplomatice ale RPR în Africa Neagră’, 7 November 1960, ANIC [Arhivele Naționale Istorice Centrale], CC al PCR, Cancelarie, 47/1960 vol. II, 280–5.

4 ‘Raport asupra activității echipei de medici români detașați în Republica Guineea’, 26 July 1966, MSPS, S2, 1966, vol. II, 229.

5 On Albert Schweitzer's ambivalence between a colonial, white supremacist outlook and his commitment to the well-being of his patients see R. Harris, ‘The Allure of Albert Schweitzer’, *History of European Ideas*, 40, 6 (2014), 804–25; R. Harris, ‘Schweitzer and Africa’, *The Historical Journal*, 59, 4 (2016), 1107–32; J. Paget, ‘Albert Schweitzer and Africa’, *Journal of Religion in Africa*, 42 (2012), 277–316.

6 J. M. Cho, ‘Provincializing Albert Schweitzer's Ethical Colonialism in Africa’, *European Legacy*, 16, 1 (2011), 80.

7 In Czechoslovakia, local personalities’ relations with Schweitzer during the interwar period and decolonization supposedly confirmed the regime's commitment to assisting former colonial peoples. In the German Democratic Republic, he epitomized anti-Nazi Germanness founded on anti-racist humanitarianism. In Prague, students from post-colonial countries criticized Schweitzer's lionization. M. Holečková, ‘The University of 17 November in Prague: Students from Third World Countries in Czechoslovakia, 1961–1974’, *Cahiers du monde russe*, 63, 3–4 (2022), 647–68; T. Suermann, ‘Albert Schweitzer and Politics’, in J. Page and M. Thate (eds) *Albert Schweitzer in Thought and Action* (Syracuse, NY 2016), 237–55.

8 Y-S. Hong, *Cold War Germany, the Third World, and the Global Humanitarian Regime* (Cambridge 2015); P. Betts, ‘Universalism and its Discontents. Humanity as a Twentieth-Century Concept’, in F. Klose and M. Thulin (eds) *Humanity. A History of European Concepts in Practice. From the Sixteen Century to the Present* (Göttingen 2016), 64–5; Č. Brković, ‘Socialist Modernist Worldmaking: Yugoslav Interventions in the International Humanitarian Debates in the 1970s’, *Humanity: An International Journal of Human Rights, Humanitarianism, and Development*, 15, 1 (2024), 18–40; S. Hearne, ‘Between Moscow and Geneva: The Soviet Red Cross and the International Red Cross Movement’, *Journal of Global History*, 20, 1 (2025), 61–81.

9 Hong, *Cold War*, 51–82 and 287–316; D. Tot, ‘Translating Humanitarian Aid into Technical Cooperation: Yugoslavia's Medical Assistance to the Algerian National Liberation Movement (1958–1965)’, *The International History Review*, 46, 6 (2024), 769–84.

10 I. Borowy, ‘East German Medical Aid to Nicaragua: The Politics of Solidarity between Biomedicine and Primary Health Care’, *História, Ciências, Saúde-Manguinhos*, 24, 2 (2017), 411–28; D. Vargha, ‘Technical Assistance and Socialist International Health: Hungary, WHO and the Korean War’, *History and Technology*, 36, 3–4 (2020), 400–17; B. C. Iacob, ‘Paradoxes of Socialist Solidarity: Romanian and Czechoslovak Medical Teams in North Korea and Vietnam (1951–1962)’, *Monde(s). Histoire, Espaces, Relations*, 20, 2 (2021), 117–40.

11 I. Borowy, ‘Health-Related Activities of the German Democratic Republic in West Africa during the 1960s’, in P. Muehlenbeck and N. Telepneva (eds), *Warsaw Pact Intervention in the Third World Aid and Influence in the Cold War* (I. B. Tauris 2018), 173–96; B.C. Iacob, ‘Health’, in J. Mark and P. Betts (eds), *Socialism Goes Global: The Soviet Union and Eastern Europe in the Age of Decolonization* (Oxford 2022), 254–86; P. Michaels, ‘Soviet Medical Internationalism amid Destalinization, 1953–1958’, *The Soviet and Post-Soviet Review*, 50 (2023), 40–63.

12 E. Manela, ‘A Pox on Your Narrative: Writing Disease Control into Cold War History’, *Diplomatic History*, 34, 2 (2010): 299–323; A-E. Birn and N. Krementsov, ‘“Socialising” Primary Care? The Soviet Union, WHO and the 1978 Alma-Ata Conference’, *BMJ Glob Health*, 3, Suppl. 3 (2018): 1–15; D. Vargha, ‘The Socialist World in Global Polio Eradication’, *Revue d’études comparatives Est-Ouest*, 1, 1 (2018): 71–94; B.C. Iacob, ‘Malariology and Decolonization: Eastern European Experts from the League of Nations to the World Health Organization’, *Journal of Global History*, 17, 2 (2022), 233–53.

13 The case of the Soviet Union stands apart: authors convincingly discussed the role of medicine in the Bolshevik colonization of ‘backward’ regions in the USSR, particularly Central Asia. P. Michaels, ‘Medical Propaganda and Cultural Revolution in Soviet Kazakhstan, 1928–41’, *Russian Review*, 59 (2000), 159–78; P. Michaels, *Curative Powers: Medicine and Empire in Stalin's Central Asia* (Pittsburgh, PA 2003); S. Jones and A. Amramina, ‘Entangled Histories of Plague Ecology in Russia and the USSR’, *History and Philosophy of the Life Sciences*, 40, 3 (2018), 1–21; M. Eby, ‘Vectors of Socialism. Epidemics and the Practice of Soviet Power in Central Asia, 1920–1941’, Ph.D. Thesis, New York University (2025).

14 Baker et al. (eds), *Off White*. Also I. Law, *Red Racisms. Racism in Communist and Post-Communist Contexts Mapping* (New York 2012); I. Kalmar, *White But Not Quite: Central Europe's Illiberal Revolt* (Bristol 2022); J. Mark, ‘Race’, in Mark and Betts (eds), *Socialism*, 221–54.

15 C. Baker, *Race and the Yugoslav Region: Postsocialist, Post-conflict, Postcolonial?* (Manchester 2018); M. Turda and M.S. Quine, *Historicizing Race* (London 2018); A. Antić, *Non-Aligned Psychiatry in the Cold War. Revolution, Emancipation and Re-Imagining the Human Psyche* (London 2021); A. Parvulescu and M. Boatcă, *Creolizing the Modern: Transylvania Across Empires* (Ithaca 2022); B. Balogun, *Race and the Colour-Line. The Boundaries of Europeanness in Poland* (London 2024).

16 M. Todorova, *Unequal under Socialism: Race, Women, and Transnationalism in Bulgaria* (Toronto 2021), 16 and 60.

17 C. Mills, *The Racial Contract* (Ithaca, NY 1997). On race as ‘a system of meaning’ see S. Hall, *The Fateful Triangle: Race, Ethnicity, Nation* (Cambridge 2017), 33.

18 E. Dragomir, ‘The Perceived Threat of Hegemonism in Romania during the Second Détente’, Cold War History, 12, 1 (2012), 111–34; E. Dragomir, *Pericolul sovietic. Percepții și relații româno-sovietice în România postbelică* (București 2019).

19 M. Tudor, ‘Humanitarianism and the Global Cold War, 1945–1991’, in S. Roth, B. Purkayastha and T. Denskus (eds), *Handbook on Humanitarianism and Inequality* (Cheltenham Glos 2024), 40.

20 M. Barnett, *Empire of Humanity. A History of Humanitarianism* (Ithaca, NY 2011), 13; K. O'Sullivan, ‘Humanitarian Worldmaking in the Age of Decolonisation: Afterword’, *International History Review*, 46, 6 (2024), 825–6.

21 ‘Popoarele coloniale au dreptul la viață și sănătate’, *Muncitorul sanitar*, 18 February 1961, 4.

22 O. Berlogea, ‘Sănătatea popoarelor’, *Muncitorul sanitar*, 4 April 1958, 4.

23 C. Mills, ‘The Chronopolitics of Racial Time’, *Time and Society*, 29, 2 (2020), 313.

24 ‘Greaua situaţie sanitară în Congo’, *Muncitorul sanitar*, 3 September 1960, 4.

25 T. Vornicu, ‘De la “daba” spre kilowații fluviului Konuré’, *Scînteia*, 28 January 1961, 3

26 ‘Greaua’, 4; ‘Popoarele’, 4.

27 M. Liviu, ‘Dr. Albert Schweitzer la 85 de ani’, *Magazin*, 145 (1960), 8.

28 M. M., ‘Albert Schweitzer’, *Lumea*, 2 (1964), 31.

29 Official Records WHO, no. 111, Part II, 170 and 316.

30 Nota 176/3, 24 December 1960, MAE [Ministerul Afacerilor Externe]/Guinea 25/1961, 7–13.

31 Telegramă ambasada RPR în Conakry, 7 February 1963, MAE/Guineea 70/1962, 1.

32 ‘Notă: unele aspecte ale relațiilor R.S.Cehoslovace cu țările din Africa și Orientul Mijlociu’, MAE/Africa 82/1966, 56.

33 P. Muehlenbeck, *Czechoslovakia in Africa, 1945–68* (London 2015); B. Buzássyová, ‘Socialist Internationalism in Practice: Shifting Patterns of the Czechoslovak Educational Aid Programmes to Sub- Saharan Africa, 1961–1989’, PhD thesis, Faculty of Arts of Comenius University (2021).

34 N. Radonjić, ‘A Non-Aligned Continent: Africa in the Global Imaginary of Socialist Yugoslavia’, in P. Stubbs (ed), *Socialist Yugoslavia and the Non-Aligned Movement. Social, Cultural, Political, and Economic Imaginaries* (Montreal 2023), 303.

35 ‘Informare privind vizita delegației de stat a R.P.România în R.S.F.Iugoslavia (22–30 noiembrie 1963)’, ANIC, CC al PCR, Relații Externe, 7I, 159.

36 ‘Raport privind vizita delegației de partid și guvernamentale a R.P.România în Algeria, 30 octombrie-6 noiembrie 1964’, ANIC, CC al PCR, Cancelarie, 104/1964, 2 and 10.

37 D. Tot, ‘Long-term Investment or Short-term Profit? The Divergence of Views on Technical Assistance to Algeria Between the Yugoslav Authorities and Self-managing Enterprises’, *Comparativ*, 33, 3 (2023), 401.

38 ‘Notă: relațiile RSR cu țăriile africane’, 30 July 1966, MAE/Africa 83/1966, 30. B. Ranteș, *Relațiile României cu state din Africa Ecuatorială și de Vest, 1960–1974* (Târgoviște 2017).

39 ‘Declarație cu privire la poziția Partidului Muncitoresc Român în problemele mișcării comuniste și muncitorești internaționale adoptată de Plenara lărgită a CC al PMR din aprilie 1964’, *Scînteia*, 26 April 1964; ‘Cuvântarea tovarășului Gheorghe Gheorghiu-Dej la conferința organizației de partid a orașului București’, *Scînteia*, 16 February 1964.

40 T. Roșca, ‘Informare asupra vizitei în Forécariah’, 3 February 1966, MSPS Secretariat Relații 1966 vol. II, 275; ‘Notă de convorbire’, 27 October 1964, ANIC, CC al PCR, Relații externe, 7/1974, 24–6.

41 A. Jersild, ‘Sino-Soviet Rivalry in Guinea-Conakry, 1956–1965’, in P. Babiracki and A. Jersild (eds), *Socialist Internationalism in the Cold War. Exploring the Second World* (London 2016), 309.

42 E. Gall, ‘Albert Schweitzer. O personalitate complexă, contradictorie și controversată’, *Contemporanul*, 6 December 1965, 8.

43 ‘Referat acordare ajutor tehnic medical Republicii Guineea’, MSPS, S2, 1964, vol. II, 25; ‘Asistența tehnică în domeniul medical acordată de către R.S.România’, 6 April 1966, MAE/Guinea 170/1965, 3–4.

44 ‘Raport asupra activității’, 225.

45 Jersild, ‘Sino-Soviet’, 314. On the features of Chinese medical humanitarianism in Africa during the 1960s see D. Zou, ‘Socialist Medicine and Maoist Humanitarianism: Chinese Medical Missions to Algeria, 1963–1984’, PhD Dissertation, Columbia University (2019).

46 H. Liman, ‘Starea sănătății lumii’, *Scînteia*, 24 June 1967, 6.

47 D. Bannister, ‘Public Health and its Contexts in Northern Ghana, 1900-2000’, PhD thesis, SOAS University of London (2017), 132–98.

48 O. Owusu-Afriyie, ‘Cerere de doctori pentru serviciul medical ghanez’, MSPS, Secretariat, 1965 vol. I, 305–4.

49 V. Marinescu to C. Mănescu, 14 May 1965, MAE/Ghana 157/1965, 1.

50 B.C. Iacob, ‘A Babel in Bucharest: “Third World” Students in Romania, 1960s–1980s’, *Cahiers du monde russe*, 63, 4 (2022), 683–4.

51 ‘Informare privind consfătuirea miniștrilor sănătății din țările socialiste, 5–14 septembrie 1961’, ANIC, CC al PCR, Cancelarie 86/1961, 5; ‘Organizarea asistenței medicale în mediul rural’, 17 September 1959, MSPS, S2, 1959–60, vol. I, 13–2.

52 Mark, ‘Race’, 227.

53 I am drawing here on David Arnold's work on ‘inventing tropicality’, in *Colonizing the Body: State Medicine and Epidemic Disease in Nineteenth-century India* (Los Angeles 1993), 42–3 and *The Problem of Nature: Environment, Culture and European Expansion* (Hoboken, NJ 1996), 142.

54 I. Popescu către MAE, 20 November 1965, MAE/Guineea 170/1965, 31.

55 ‘Informare privind condițiile de viață și de muncă ale medicilor români din Guineea’, 25 February 1966, MSPS, S2, 1966, vol. I, 280–79.

56 Adresă MSPS către MAE, 11 May 1965, MSPS, S2, 1965, vol. I, 346.

57 ‘Referat’, 26 August 1966, MSPS Secretariat Relatii 1966 vol. II, 87–5.

58 MSPS, Schimb de experiență-Diverse delegări în străinătate, 1961, 72–60; MSPS Rapoarte Delegații 1963, 132–1.

59 ‘Informare privind unele aspecte ale situației social-culturale din Guineea’, 22 April 1966, MAE/Guinea 201/1966, 85.

60 A. Inandolo, *Arrested Development. The Soviet Union in Ghana, Guinea, and Mali, 1955–1968* (Ithaca, NJ 2022). Also, Sara Lorenzini, *Global Development: A Cold War History* (Princeton, 2019).

61 MAE/Guinea, 177/1965, 104.

62 ‘Raport asupra activității’, 226–5.

63 ‘Scrisoare a directorului Direcției Relațiilor Culturale către MSPS legată de raportul medicilor din Guineea’, 25 July 1966, MSPS, S2, 1966, vol. II, 230

64 ‘Raport asupra activității’, 228–6.

65 ‘Raport asupra activității’, 226 and 224.

66 E. Burton, Z. Ginelli, J. Mark and N. Radonjić, ‘The Travelogue: Imagining Spaces of Encounter—Travel Writing Between the Colonial and the Anti-Colonial in Socialist Eastern Europe, 1949–1989’, in K. Roth-Ey (ed), *Socialist Internationalism and the Gritty Politics of the Particular. Second-Third World Spaces in the Cold War* (London 2023), 254.

67 ‘Raport asupra activității’, 225. Among the WHO experts dispatched to Guinea, there were two Romanians: Nicolae Cajal, who coordinated an anti-poliomyelitis vaccination study; and G. Iacob, who, between 1970 and 1975, headed the WHO-Guinea program for basic health care services. See various reports in country folders WHO3/102; WHO022/AFRO/GUI/003, WHO022/AFRO/GUI/004.

68 A. Jersild, ‘Socialist Advisers and the Dilemmas of the “Socialist World System”: Sino-Soviet Exchange as a Model for Failure in Guinea-Conakry, 1950–64’, *European Review of History*, 30, 3 (2023), 442.

69 W. Anderson, *Colonial Pathologies: American Tropical Medicine, Race and Hygiene in the Philippines* (Durham, NC 2006), 183.

70 ‘Raport asupra activității’, 226.

71 Bologa, ‘Albert’, 12.

72 ‘Raport asupra activității’, 226–5.

73 ‘Informare privind conditiile”, 282.

74 D. Neill, *Networks in Tropical Medicine: Internationalism, Colonialism, and the Rise of a Medical Specialty* (Redwood City, CA 2012), 49.

75 Roșca, ‘Informare asupra vizitei’, 275.

76 S. Mazov, *A Distant Front in the Cold War: The USSR in West Africa and the Congo, 1956–1964* (Washington DC 2010), 189. On the Guinea leadership's modernist anxieties about backwardness see M. McGovern, *Unmasking the State: Making Guinea Modern* (Chicago 2013).

77 Roșca, ‘Informare’, 277.

78 E. Montestruc, ‘Rapport sur l’évaluation de la campagne de masse contre le lèpre en Guinée, 26 juin-3 juillet 1965’ and Dominique Martinez to M. Blanc, 3 December 1965 in WHO Guinea 3-Leprosy [microfilms].

79 C. Ionescu, ‘La un institut african de seruri’, *Muncitorul sanitar,* 11 May 1966, 4.

80 WHO022/AFRO/GUI/018, 9.

81 ‘Raport asupra activității’, 225; ‘Adresă MAE către MSPS’, 8 December 1965, MSPS, Secretariat, 1965 vol. II, 224; D. Stănescu, ‘Informare grup specialiști români’, 21 February 1966, MAE/Guineea 201/1966, 4.

82 Paget, ‘Albert’, 290; Cho, ‘Provincializing’, 80.

83 Y. Gradskova, *The Women's International Democratic Federation, the Global South and the Cold War: Defending the Rights of Women of the ‘Whole World’?* (London 2021), 121–4.

84 C. Corbu, ‘Seminarul “Ocrotirea mamei şi copilului”. O problemă a secolului’, *Contemporanul*, 18 November 1960, 3.

85 ‘Seminar “Ocrotirea mamei şi a copilului”’, *Muncitorul Sanitar*, 19 November 1960, 3.

86 C. Doboș, ‘Disciplining Births: Population Research and Politics in Communist Romania’, *The History of the Family*, 25, 4 (2020), 603–7.

87 ‘Sub semnul prieteniei și solidarității’, *Femeia*, 1960, 4. On positive eugenics as central to (post)colonial development see C. Decker and E. McMahon, *The Idea of Development in Africa. A History* (Cambridge 2020), 70–7. On Marinescu and eugenics see Marius Turda, *În căutarea românului perfect: specific național, degenerare rasială și selecție socială în România modernă* (Iași 2024), 166 and 179.

88 ‘Referat’, 25 November 1960, IRRCS, 9/1960–2, 35.

89 ‘Notă MSPS’, 4 March 1966, MSPS, S2, 1965, vol. III, 207 and 218; N. Coja, ‘Vidextractia obstetrical’ă', *Muncitorul sanitar*, 18 April 1964, 2.

90 ‘Continentul african și eradicarea malariei. Interviu cu Gh. Lupașcu’, *Muncitorul sanitar*, 4 December 1965, 4; N. Georgescu, ‘Eradicarea malariei în județul Tulcea’, in Gheorghe Brătescu (ed), *Momente din trecutul medicinii* (București, 1983), 845–53.

91 G. Lupaşcu, ‘Prin Senegal’, *Lumea*, 6 January 1966, 21.

92 G. Lupașcu, ‘Report on the Application of Malaria Chemotherapy and Chemoprophylaxis in certain African Countries’, WHO/Mal/Exp.Com.12/WP/6.65, 29 and 40.

93 J. Webb, *The Long Struggle Against Malaria in Tropical Africa* (Cambridge 2014), 70–88.

94 Lupașcu, ‘Report’, 38–40.

95 Lupaşcu, ‘Prin Senegal’, 21.

96 ‘Semnificația unei scrisori. Interviu cu Gheorghe Lupașcu’, *România liberă*, 23 December 1966, 3.

97 I am drawing on R. Pailey, ‘De-centring the “White Gaze” of Development’, *Development and Change*, 51, 3 (2019): 733.

